# Psychiatric Home Hospitalization: The Role of Mental Health Nurses—A Scoping Review

**DOI:** 10.3390/healthcare13030231

**Published:** 2025-01-24

**Authors:** Marisa Soares, Vânia Martins, Margarida Tomás, Luís Sousa, Tiago Nascimento, Patrícia Costa, Graça Quaresma, Pedro Lucas

**Affiliations:** 1 Research and Innovation Knowledge Center (CCII), Local Health Unit Almada-Seixal, Psychiatry and Mental Health Service, 2805-267 Almada, Portugal; marisasoares82@gmail.com (M.S.); vania@arribaclub.com (V.M.); 2 Nursing Research, Innovation and Development Centre of Lisbon (CIDNUR), Nursing School of Lisbon, 1600-190 Lisboa, Portugal; matomas@esel.pt (M.T.); luismmsousa@gmail.com (L.S.); tnascimento@esel.pt (T.N.); patriciacosta@esel.pt (P.C.); mgquaresma@esel.pt (G.Q.); 3 Comprehensive Health Research Centre (CHRC), University of Évora, 7000-801 Évora, Portugal; 4 Atlântica Health School, 2730-036 Barcarena, Portugal

**Keywords:** nurse’s role, home hospitalization, psychiatry and mental health, management, review

## Abstract

The lack of evidence synthesis studies on the role of psychiatric mental health nursing in the context of psychiatric home hospitalization underscores the limited research on nurses providing care in these teams, particularly concerning their experiences and responsibilities and the actual role of nurses in this context. This knowledge has the potential to improve the quality of mental healthcare by guiding nursing practices. **Objective**: To map the concepts related to the role of mental health nurses in psychiatric home hospitalization. **Methods**: A scoping review was conducted using the methodology proposed by the JBI, involving five methodological stages. No search limits were applied except for language. **Results**: The review identified five key dimensions of nursing in psychiatric home hospitalization: satisfaction; care models; the therapeutic relationship; the care environment; the organization of care. These interconnected dimensions influence quality care. Satisfaction among nurses, patients, and families is associated with improved outcomes and reduced stigma. The therapeutic relationship is characterized by a humanistic approach, emphasizing dialogue, empathy, and shared decision making. Additionally, the importance of care organization is highlighted, including individualized care plans, medication management, and intersectoral collaboration. **Discussion**: The five nursing dimensions align with the Quality Standards of the Portuguese Nursing Council and are consistent with the scientific literature. **Conclusions**: Although there is a shortage of studies on this topic, this review allows for the synthesis of nursing interventions and reflection on the paradigm shift in care within the scope of psychiatric mental health nursing interventions. Future studies highlighting the value of mental health nursing interventions, with a particular focus on nursing-sensitive indicators and employing quantitative or mixed methods, will be crucial to furthering the analysis conducted thus far.

## 1. Introduction

Home hospitalization (HH), as a model of care delivery in the home, presents itself as an alternative to conventional hospital admission, offering continuous care at home for patients who meet specific clinical, social, and geographical criteria, allowing them to be monitored by a Home Hospitalization Unit, with the consent of both the patient and their family. This model contributes to optimizing bed management within the National Health Service in Portugal [1]. This approach strengthens the humanization of care for patients, families, and healthcare professionals, fostering a sense of personal connection within the family environment. Moreover, it allows for the better management of available beds for treating patients with acute illnesses within the Portuguese NHS.

Despite the increased awareness of mental health concerns in the media and on social networks, particularly during the COVID-19 pandemic, psychiatric hospitalization remains largely outside the public eye, maintaining its stigmatizing identity, which can lead to discriminatory implications when individuals reveal their hospitalization upon returning to daily life [2]. Additionally, hospitalization can lead to feelings of disruption for individuals due to the physical distance from their familiar surroundings for a period, after which they return under a regimen of medication, whose iatrogenic effects may hinder their ability to fully engage in life [2,3]. In this sense, the literature describes returning home and resuming daily life after psychiatric discharge as a challenging and complex process [4,5]. Individuals must cope with the symbolic burden of psychiatric hospitalization, which not only reflects their inability to remain in the community—undermining their sense of autonomy and self-efficacy—but also forces them to face the stigma and discrimination still present in society, stemming from psychiatric hospitalization and the resulting mental health diagnosis [4]. There are also negative outcomes of hospitalization in psychiatric services that must be considered, such as readmission, suicide, the risk of aggression, stigma surrounding mental illness, and a reduction in self-control over one’s life [6], especially when there is a history of multiple admissions or long-term stays [7,8].

To mitigate this issue, teams known as crisis resolution and home treatment teams (CRHTTs) in mental health have emerged as an alternative to hospital-based psychiatric care. These teams were developed in the mid-1990s in various countries, including the United Kingdom, the United States, Australia, Norway, Belgium, Germany, Spain, Canada, and the Republic of Ireland [9,10,11]. These countries have published studies on the implementation of CRHTTs, reporting a reduction in hospital admissions and treatment costs, as well as higher levels of satisfaction among those who accessed this type of service, compared to those who received standard hospital care [11]. In Portugal, home hospitalization (HH) in psychiatry is not yet adopted practice, despite having existed for around 20 years in some European countries. Although the HH model is recognized as a viable alternative with the potential to improve mental healthcare, it has not yet been established as a priority in the Portuguese Health System.

CRHTTs thus provide intensive mental healthcare to individuals experiencing a mental health crisis who would otherwise require hospitalization, allowing them to receive treatment at home [12]. Numerous studies today confirm the effectiveness of this care model [7,11,13]. However, research on the role of nursing care in this context remains scarce, particularly regarding the experiences, responsibilities, and interventions of nurses [14,15].

Mental health nursing, particularly within the context of psychiatric HH (PHH), is multidimensional and complex. It must promote health, dignity, and self-determination, ensuring that effective practices are implemented by competent, confident teams aligned with patients’ needs [16]. Valuing nurses’ experiences can shift nursing paradigms from biomedical models to holistic recovery perspectives, enhancing the quality of care. Moreover, nurses’ satisfaction with the care they provide has a positive impact on patients, as their satisfaction is a crucial indicator of service effectiveness and quality [17,18].

The aim of this review is to map the concepts related to the role of mental health nurses in psychiatric home hospitalization, with a particular focus on identifying the activities and interventions performed, exploring nurses’ perceptions and experiences, and assessing the impact of nursing care on patients receiving home-based care and their families. This study, therefore, seeks to contribute to the development of the nursing profession by highlighting what patients with mental illness and their families value most in the care and interactions with nurses in the home setting, emphasizing the importance of person-centered care.

## 2. Materials and Methods

Given the complexity of understanding the role of nursing in the context of psychiatric home hospitalization, as evidenced in the literature, a scoping review methodology was selected as the most appropriate approach for synthesizing the knowledge. This scoping review was conducted following the methodology proposed by the JBI [19] and aligned with the Preferred Reporting Items for Systematic Reviews and Meta-Analyses extension for scoping reviews (PRISMA-ScR) [20]. A preliminary search was performed in MEDLINE, CINAHL, the Cochrane Database of Systematic Reviews, and JBI Evidence Synthesis, and no ongoing scoping reviews on this topic were identified. The study protocol was developed and registered on the Open Science Framework (https://doi.org/10.17605/OSF.IO/SD9ZG).

The five methodological stages recommended by the JBI (2015) for scoping reviews were followed: (1) identifying the research question, (2) identifying relevant studies, (3) selecting studies, (4) charting data, and (5) summarizing and synthesizing the results.

The following research question (stage 1) was established using the PCC mnemonic (Population, Concept, Context): How is the role of nurses in psychiatric home hospitalization characterized in scientific literature?

To identify relevant studies (stage 2), a search was conducted using the EBSCOhost platform, Nursing Reference Centre Plus, and JBI EBP Resources on Ovid. The sources used for the search included Academic Search Complete, the Cochrane Central Register of Controlled Trials, Cochrane Clinical Answers, the Cochrane Database of Systematic Reviews, the Cochrane Methodology Register, CINAHL Complete, MEDLINE Complete, the Psychology and Behavioral Sciences Collection, and Medic Latina. The articles obtained are limited to the period 2016 to 2023. Database selection for this review was guided by institutional accessibility and relevance to the field of mental health nursing. While databases such as Scopus and Web of Science were not included, we relied on complementary sources (CINAHL, MEDLINE, Psychology and Behavioral Sciences Collection) that are recognized for their comprehensiveness in the health sciences literature.

The search strategy utilized the PCC mnemonic, as recommended by the JBI for scoping reviews. This represented the terms for Population, Concept, and Context, which were defined and combined using Boolean operators, wildcards, and truncation. An initial limited search was conducted in CINAHL and MEDLINE to identify articles on the topic and examine titles, abstracts, keywords, or indexed terms to inform the development of the search strategy for the second stage. Based on this initial search, the following were determined:

- Population: studies involving nurses using the natural terms Nursing OR Nurse OR Nurse* Manager* OR Nursing Management*;
- Concept: literature addressing the topic of home hospitalization using the natural terms Home Treatment Team OR Home Hospitalization;
- Context: studies limited to the context of mental health and psychiatry using the natural terms Mental Health OR Psychiatric.

No cultural or geographical restrictions were applied, and the search terms were comprehensive, with no limitations. Studies in Portuguese, Spanish, and English were considered.

The selected studies included the role and nursing interventions in home treatment teams operating in mental health and psychiatric contexts. Due to the exploratory nature of this review and with the aim of providing an overview of the current evidence, study design and methodological quality were not used as selection criteria. Studies were excluded if they did not align with the concept and context or if they did not include nurses as part of the study. Specific exclusions included studies that did not involve nurses in clinical practice, that did not address nursing roles in home psychiatric hospitalization, and that did not focus on mental health or psychiatry. Studies involving student nurses were also excluded. Articles that were not in English, Portuguese, or Spanish were also excluded.

**Figure 1:**
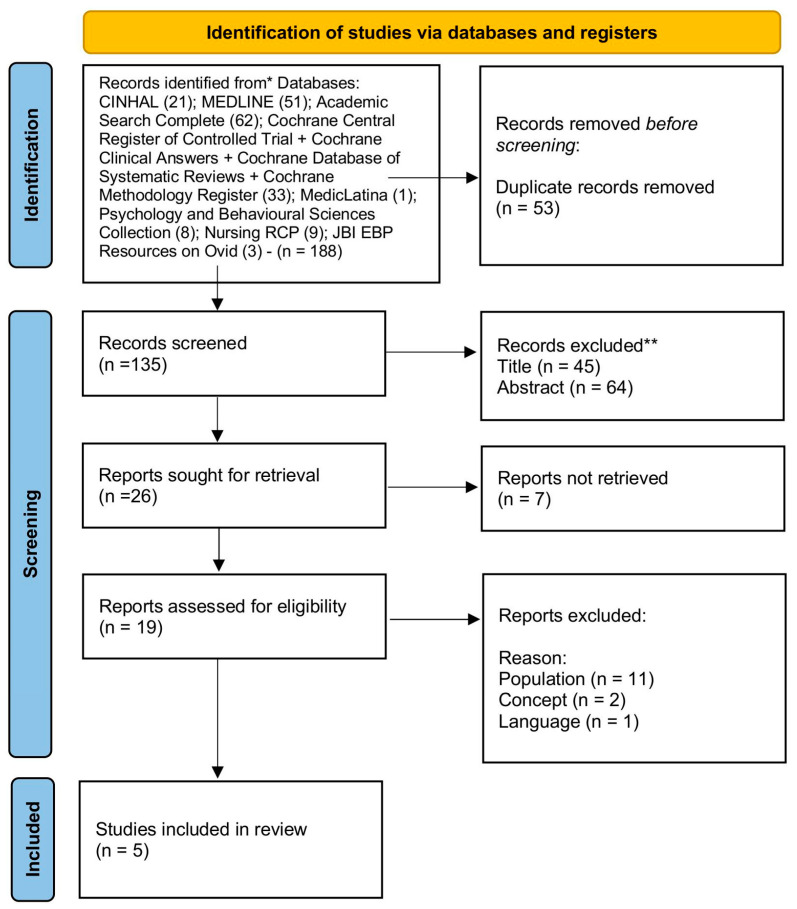
PRISMA 2020 flow diagram. * Consider, if feasible to do so, reporting the number of records identified from each database or register searched (rather than the total number across all databases/registers). ** If automation tools were used, indicate how many records were excluded by a human and how many were excluded by automation tools.

For the selection of studies (stage 3), all articles were independently reviewed by the researchers, starting with titles and abstracts, based on the inclusion and exclusion criteria. Out of the initial 188 articles, 53 were excluded as duplicates. After removing duplicates, the study selection process was conducted in three screening stages: (1) by title, (2) by abstract, and (3) by full text. In the first screening stage, 45 articles were excluded after reviewing the titles, leaving 90 articles for abstract review. In the second screening stage, 64 articles were excluded based on their abstracts. A total of 26 articles were fully reviewed, of which 7 were inaccessible, even after library searches. Thus, 19 articles were fully analyzed, with an additional 2 articles included after reviewing references (gray literature). In total, 16 articles were excluded, and 5 were selected for inclusion in the analysis. The final relevant articles reflect studies on the role and intervention of nurses in psychiatric home hospitalization teams. The PRISMA flow diagram [20] summarizes the article selection process, from identification to final inclusion. Several articles were excluded for not focusing on nursing or home hospitalization teams (Figure 1).

Based on variables that allowed the research questions to be addressed, data collection was conducted using a tool developed by the researchers (stage 4). The extracted variables included the author, year of publication, study title, study location, study design, sample, study objectives, results, limitations, and nursing dimensions.

In the next stage (stage 5), a data extraction table was developed from the five articles included in the study, based on the data collection instrument developed in the previous stage, which synthesized the data extracted according to the variables that addressed the research question.

## 3. Results

The evidence demonstrates a scarcity of studies focused on nurses on home hospitalization teams within the field of mental health and psychiatry. The five studies included are European, with qualitative methods being the most used. This review covers responses from a total of 23 nurses working on crisis resolution and home treatment teams (CRHTTs), 125 service users who utilized the service, and 20 family members who were also involved in the program. No literature reviews were found. Across the five studies included in this review, several dimensions of nursing were identified and categorized to synthesized concepts into the following categories: satisfaction, care models, the therapeutic relationship, the care environment, and the organization of care (Table 1).

## 4. Discussion

The aim of this review is to map the concepts related to the role of mental health nurses in psychiatric home hospitalization. This includes identifying the activities and interventions performed, exploring the perceptions and experiences of nurses, and evaluating the impact of nursing care on patients and their families.

This discussion addresses various dimensions of nursing care delivery, with a focus on key themes, such as professional and patient satisfaction, care models, therapeutic relationships, the care environment, and the service organization. These themes are interconnected, providing a holistic view of nursing practice and its impact on the quality of care, directly influencing the effectiveness and satisfaction within the mental health context. A discussion of each category follows.

### 4.1. Satisfaction

The theme of satisfaction was developed by the same author across two different studies, with two distinct perspectives: nurse satisfaction with the care provided and with home-based assistance, which had a positive impact on the quality of care and reduced patient stigma [16]; and patient and family satisfaction with the nursing care received, resulting in high levels of satisfaction with treatment [17]. Patient satisfaction is considered a key indicator in improving care quality and health service standards, making it a fundamental outcome measure. Citizens are becoming more informed and demanding, expecting high-quality standards and levels of satisfaction. The concept of satisfaction with healthcare is challenging to operationalize, as it is a multidimensional concept that considers individual aspects, past experiences, patient expectations, and dimensions such as accessibility, organization, and patient–professional interaction [21]. Ref. [18] also states that the satisfaction with a healthcare service reflects its effectiveness and quality, and patient satisfaction with nursing care is regarded as fundamental in evaluating the quality of care [22]. It is known that satisfied patients demonstrate higher adherence to therapy [23] and experience better recovery processes [24].

### 4.2. Care Model

The theme of the care model is addressed across all five articles, albeit in different ways. It consistently appears as a person-centered model, oriented towards real-world contexts and recovery, involving shared responsibilities, professional flexibility, and collaborative work [13,14,15,16,17]. This model aims to include family and social networks to achieve better outcomes. In Portugal, home hospitalization is defined as a hospital care model for providing care at home to individuals with acute or exacerbated chronic illnesses, where biological, psychological, and social conditions permit. It differs from traditional home care by offering intensive and highly specialized care for acute and complex disease states [1]. It is a model centered on patient needs and is more humanized, without the complications inherent in conventional hospitalization, offering high-quality care with clinical rigor and a holistic, humanized perspective when hospital stays are avoidable [25]. Lopes (2021) proposes a home care model aimed at refocusing care on people’s homes [26]. This model, in addition to focusing on the patient and their primary caregiver in their context, ensures the effective integration and continuity of care. In this sense, it serves as an alternative to hospital admission centered on patient needs, providing a safe, efficient, and appropriate response to illness while ensuring the continuity of care.

### 4.3. Therapeutic Relationship

The therapeutic relationship is widely described in the five articles, following a humanistic approach based on holistic understanding. It is a relationship centered on dialogue, honesty, empathy, active listening, and the absence of critical judgements. Decision making is seen as shared between professionals, patients, and families [13,14,15,16,17]. This relationship is experienced as intense, horizontal, respectful, accessible, and personalized, adapting to the environment. Patients describe positive feelings such as feeling accompanied, supported, relieved, comfortable, and understood [16,17]. It is universally defined as a flexible, available therapeutic approach based on therapeutic alliances, relational progression, and therapeutic optimism. In mental health nursing, therapeutic relationship is central to the care process, as restoring the balance of a person in mental distress relies on meaningful interpersonal relationships [27]. According to [28], the therapeutic relationship is a specific nursing care tool, centered on the uniqueness of interpersonal relationships between the nurse and the cared-for person, fostering the growth and development of both parties in a dynamic process. Ref. [29] also points out that the helping relationship is a relationship through which the nurse provides the person with the means to meet their needs. In the home care context, nursing interventions balance the relationship between nurses and patients, adapting strategies and interventions to real-world contexts and fostering deeper relational and communication skills.

### 4.4. Care Environment

The specific care environment is identified in most of the articles and is described as less invasive, restrictive, traumatic, and adverse, establishing itself as an alternative, unique space for care provision [14,16]. It also tends to reduce and overcome the social stigma associated with mental illness, being rooted in a mobile, confident approach that promotes autonomy, dignity, and self-determination. Concurrently, the high level of concern and stress experienced by professionals is evident, providing competency in risk assessment, reduction, and management, as well as ensuring patient safety, which is of the utmost importance. The home care context has always been explored by nurses for care provision, as they recognize the potential of proximity to patients and their families, valuing the establishment of interrelations and cooperation in care. This environment can simultaneously be part of both the problem and the solution in the recovery process. As such, integrating the home as a care setting ensures a realistic adjustment to the conditions in which a person lives and must recover from the acute phase of mental illness. This recovery process is inherently integrative and person-centered, involving both the individual and their family caregiver. It is also possible to relate this home care environment to the one described by Lake [30] as capable of bringing together a set of organizational characteristics in a work context that either facilitates or constrains nursing practice. Thus, the care environment is associated with professional satisfaction, the quality of the nursing care, patient safety, the effectiveness of the care provided to patients, and organizational efficiency [31].

A favorable nursing practice environment, where nurses have autonomy, control over their environment, and good relationships with the multidisciplinary team, leads to significant impacts on patient and nurse outcomes, such as lower burnout levels, increased productivity, greater professional satisfaction, and better patient outcomes in terms of care quality and safety [31,32,33,34,35,36].

### 4.5. Organization of Care

Finally, the organization of care is linked to the smooth coordination with reference services and the necessary continuity of care, as highlighted in the articles [15,17], as well as to the importance of integrated and individualized care plans, adapted to daily life activities, respecting routines and habits, and the contexts in which patients are embedded. It is also associated with various nursing interventions, particularly regarding medication management, health promotion and education, and empowerment to face and manage illness [13]. Other aspects related to care organization models, organizational functioning, nurse-to-patient ratios, and accessibility to care are also mentioned as influences on care outcomes [13,14,15,16]. As nurses are involved in all aspects of service delivery across all healthcare settings, the organization of nursing resources is essential for the organizational performance, and managers are challenged to find operational care delivery models that maximize the use of available nursing resources while ensuring safe, high-quality care [37]. In addition to understanding nursing care models and rethinking how they highlight nursing interventions, it is important to clarify the operational aspects that characterize them. Case Management (CM) is considered an individual model of “integrated care”, prioritized in the implementation of integrated, person-centered healthcare services [38]. CM requires highly qualified professionals and is used in several European countries to strengthen the role of nurses in providing and coordinating care for people with high-complexity care needs. This organization may be based on one or more working methods and is associated with various factors, such as institutional culture, nursing management, resource availability, and the characteristics of different nursing team members [39].

These five nursing dimensions identified in the studies are interlinked and aligned with the Quality Standards for Mental Health Nursing Care set by the Portuguese Nursing Council [40]. As such, they serve as an essential tool for promoting continuous improvement in specialized care and as a reference for reflecting on nursing practices. They take on greater significance in nursing management, as they aggregate specific competencies that inspire changes in work contexts, promoting significant improvements in the practice environment and the quality of care for patients.

It is also important to contextualize that autonomy, dignity and self-determination are interdependent and fundamental ethical concepts in nursing, especially in the context of PHH, reflecting a person-centered approach that respects the individuality, values and choices of the patient. The definition of autonomy appears as the ability to make free and informed decisions [41], which is essential in healthcare, while dignity is described as an inherent and intrinsic value, reinforced by respect and ethical treatment [42]. Self-determination, in turn, expands autonomy by emphasizing the ability to act according to one’s own choices, strengthening the individual’s empowerment and protagonism in their recovery [43]. In the context of this article, these concepts manifest themselves in dimensions such as the person-centered care model, the humanistic therapeutic relationship, and the less invasive and more welcoming care environment, all promoting practices that integrate dignity, autonomy, and self-determination. Thus, by recognizing dignity as the ethical basis, autonomy as the capacity to choose, and self-determination as the practical implementation of these choices, the role of nursing in PHH reinforces respect for the patient’s uniqueness and promotes the empowerment necessary for their full recovery.

Most existing studies focus on clinical, multidisciplinary, and structural aspects, with little investigation specifically into the role of nurses and their contribution to nursing care. As a quality criterion, the PRISMA-ScR checklist was rigorously applied for a structured assessment in this scoping review.

## 5. Recommendations for Future Research and Clinical Practice in Psychiatric Home Hospitalization

Future studies should deepen the knowledge about the role of nursing in PHH particularly through quantitative research that evaluates the impact of nursing care on patient recovery beyond satisfaction metrics. Exploring indicators such as the care quality, patient satisfaction, healthcare costs, and resource utilization is crucial to strengthening the evidence base for mental healthcare in home settings and promoting continuous improvement in care quality. Moreover, these efforts should aim to shift further from traditional biomedical models by adopting person-centered approaches that consider the patient’s family and social context.

This review underscores the need for research to clarify and enhance nursing interventions, allowing nurses to be recognized as agents of change and key contributors to therapeutic success in PHH. Future studies should rigorously evaluate the five dimensions of nursing identified in this review—satisfaction, care models, the therapeutic relationship, the care environment, and the care organization—while investigating their interactions and influence on patient outcomes. Comparative research is needed to assess the effectiveness of PHH compared to traditional inpatient and community care models, with a focus on long-term sustainability, including economic, logistical, and workforce considerations.

Healthcare professionals play a vital role in implementing person-centered, recovery-oriented care models that actively involve patients and their families. Strong therapeutic relationships based on trust, respect, and empathy must be prioritized while ensuring that home care environments are safe, therapeutic, and conducive to patient autonomy and dignity. Optimizing care organization through integrated, individualized care plans and effective collaboration among team members and referral services is also essential. To support these initiatives, continuous professional training is critical to developing the competencies required for mental healthcare delivery in home settings.

By addressing these recommendations, researchers and healthcare professionals can contribute to advancing the scientific knowledge on PHH, improving care quality, and ensuring that research findings are effectively translated into clinical practice. Collaborative efforts between researchers and practitioners are fundamental to achieving these goals and sustaining innovative approaches in psychiatric home hospitalization.

## 6. Limitations of the Study

This review has some limitations that deserve to be highlighted for a more complete understanding of its scope and implications: A lack of studies on the role of nursing in psychiatric home hospitalization: One of the main limitations is the notable lack of studies that specifically investigate the role of nurses in the context of psychiatric home hospitalization. Most of the existing research focuses on clinical, multidisciplinary, and structural aspects, neglecting the specific contribution of nursing care. Limited focus on quantitative indicators: This review points to the absence of studies with quantitative methodologies, which allow the impact of nursing interventions to be assessed in an objective and measurable way. Most of the studies included used qualitative methods, which limit the ability to establish cause-and-effect relationships and measure the effectiveness of interventions. The lack of nursing-sensitive indicators makes it difficult to assess the impact of care on patient recovery beyond satisfaction. Small and homogeneous samples: Some of the included studies had small and specific sample sizes, which limits the generalizability of the results to other populations or settings. Difficulty in isolating the nursing perspective: In some studies, there is difficulty in isolating the nursing perspective within a service that includes other perspectives of care. This can lead to overlapping information and less clarity about the specific role of nurses. Language restriction: Although no explicit cultural or geographic limitations were applied to this study, the inclusion criteria were restricted to articles published in Portuguese, Spanish, and English. This decision was based on the proficiency of the research team in these languages and the limited resources available for translation. Although this approach was intended to ensure the accurate interpretation of the results, it inherently introduces potential bias by excluding studies published in other languages. This linguistic restriction may have implications for the cultural and geographic diversity of the results, which should be considered when interpreting the findings. Possible bias in satisfaction responses: Studies assessing patient and family satisfaction may have bias in the responses, as participants may feel pressure to provide “correct” or positive responses. This limitation highlights the need to use other assessment tools to complement satisfaction-based outcomes. The geographic restriction of the included studies: The studies selected for this review are predominantly European, which limits the applicability of the results to other geographic and cultural contexts. Nursing practices can vary significantly across different health systems and cultures, so the results may not be directly transferable to other regions. A lack of literature reviews: This review found that there were no literature reviews on the topic. The lack of previous reviews not only demonstrates the novelty and importance of the topic but also highlights the need for more syntheses to consolidate the knowledge in the area. Limitations of individual studies: in addition to the general limitations of this review, the individual studies included also have their own limitations. Difficulty in accessing some studies: In the process of selecting the studies, some articles were considered inaccessible, which may have introduced bias in the results. Despite efforts to retrieve them, their absence may have limited the scope of this review.

## 7. Conclusions

This review identified a significant shortage of studies addressing the role of mental health and psychiatric nursing specifically in the context of psychiatric home hospitalization. This care model, despite having been implemented for around 20 years in some European countries, has not yet established itself as a priority. Most of the existing studies focus on clinical, multidisciplinary, and structural aspects, with few investigating the role of nurses and the contribution of nursing care. This gap makes it difficult to clearly understand the available evidence.

However, this review identified that PHH represents a paradigm shift in mental health treatment, with a focus on person-centered care, beyond institutional boundaries. The results reinforce the importance of the role of the nurse as a key factor in therapeutic success, promoting trust and facilitating patient recovery in this context.

The main nursing dimensions identified in this review are as follows:

Satisfaction: Both nurses and patients and families demonstrate high levels of satisfaction with the care provided in the home environment, which contributes to improved quality of care and reduced stigma. Patient satisfaction is a crucial indicator of the quality and effectiveness of the service;

Care model: Studies point to a person-centered, recovery-oriented model with shared responsibilities and professional flexibility. This model aims to involve the family and social networks, providing more humanized care adapted to the patient’s needs;

Therapeutic relationship: The therapeutic relationship is described as a central element in care, based on dialogue, empathy, and active listening, promoting an environment of support and trust. Establishing a strong therapeutic relationship between the nurse and the patient is fundamental to recovery;

Care environment: The home environment is considered less invasive and restrictive, promoting the patient’s autonomy, dignity, and self-determination. This environment can be both part of the problem and part of the solution, but integrating the home into the treatment process allows for a more realistic recovery;

Organization of care: The organization of care highlights the need for individualized and integrated care plans that are adapted to the patient’s daily life and that respect their routines and habits. Medication management, health promotion, and empowerment to cope with the disease are also crucial components of the organization of care.

In terms of the structure and organization of nursing care and interventions, this review was essential to map and organize concepts and dimensions of clinical nursing practice in mental health.

In conclusion, this review underscores the transformative potential of psychiatric home hospitalization as a care model that prioritizes humanized, person-centered approaches while addressing the complexities of mental healthcare beyond institutional boundaries. By highlighting the pivotal role of nurses in fostering therapeutic relationships, empowering patients, and delivering individualized care, this study emphasizes the urgent need for the further exploration of nursing-specific contributions in this field. Bridging the existing gaps in the knowledge through future research can not only solidify the evidence base for psychiatric home hospitalization but also inspire innovative practices that elevate the quality of care, promote patient autonomy and dignity, and ultimately transform mental health services into more inclusive, flexible, and recovery-oriented systems.

## Figures and Tables

**Table 1:** Data extraction table [13,14,15,16,17].

Authors, Year, City, Country, Title, Journal	Study Design, Sample	Study Objectives	Results	Limitations	Nursing Dimensions
Vázquez, 2023, Barcelona, Spain, Accompanying Mental Health Problems at Home: Preliminary Data from a Crisis Resolution and Home Treatment Team in Catalonia, *Journal of Psychiatric and Mental Health Nursing*, 30	Quantitative and Descriptive Study Sample: 105 participants representing the total number of individuals admitted and discharged from a CRHTT between November 2017 and December 2019. Assessment scales used included the Health of the Nation Outcome Scale (HoNOS) and the Global Assessment of Functioning (GAF) scale at admission and discharge.	To share the organizational functioning, therapeutic approach, and outcomes of a CRHTT in Catalonia, Spain; to provide a description of the CRHTT, including sociodemographic data and the main diagnoses of individuals assisted, referral sources, and discharge referrals; to compare mental health assessments at admission and discharge, as well as the total number of psychiatric hospital admissions in the same health area in the years before the CRHTT began and in the two years following its implementation.	At discharge, a statistically significant improvement was found in the overall GAF and HoNOS scores, as well as in all subscales. The total number of psychiatric admissions was 375 in 2016 and 2017, compared to 339 in 2018 and 2019.	The data provided are limited and only allow for the generation of working hypotheses for future research.	1. Professional stability; 2. Clinical improvement in symptoms and functionality, as well as crisis resolution without the need for hospital care in most cases; 3. Smooth coordination with community and hospital reference services; 4. Accessibility to the program; 5. Flexibility to adapt the intervention time to actual needs; 6. Care based on a horizontal and empathetic approach, dialogue, and shared decision making.
Giménez-Díez, 2022, Barcelona, Spain, Nurses’ Experiences of Care at Crisis Resolution Home Treatment Teams: Case study research, *Journal of Psychiatric and Mental Health Nursing*, 29	Case Study Sample: 10 nurses. No exclusion criteria. Convenience sampling. Data collection: Semi-structured interviews based on a literature review. A pre-test was applied. Procedure: Ten in-depth, semi-structured qualitative interviews were conducted. Each interview lasted one hour and was audio-recorded and transcribed.	To understand the experiences of mental health nurses working on crisis resolution home treatment teams. Case studies provide researchers with the opportunity to gain an in-depth understanding of the conditions, thoughts, actions, interventions, and environments of the individuals involved.	Three main categories emerged from the data analysis to understand the nurses’ perceptions and interpretations of care in CRHTT services: nurses’ perspectives on the care provided, the home nursing care environment, and the home nursing care plan.	The CRHTT sample was based on urban, city-center areas, which limits the generalizability of the findings. The study could have benefited from the use of other data collection methods to triangulate the data. The sample size was insufficient or biased, making the study difficult to replicate in other contexts.	1. Nurses’ perspectives on the care provided: the act of caregiving; nurse satisfaction with the care provided; 2. Nursing care environment: patient environment; therapeutic relationship; 3. Home nursing care plan: nursing diagnoses, interventions, and outcomes.
Giménez-Díez, 2020, Barcelona, Spain, Treating mental health crises at home: Patient satisfaction with home nursing care, *Journal of Psychiatric and Mental Health Nursing*, 27	Mixed Methodology The population sample consisted of patients and their family members (each participating family member was associated with a patient) who were treated by the CRHTT for two months in 2018. Inclusion criteria: participants received at least three weeks of home-based treatment. The total sample included in the study was 20 patients and 20 family members.	The objective was to evaluate the satisfaction of patients and their families with the nursing care provided through a home care program, aiming to accurately capture the experiences of the interviewees regarding the home hospitalization process and the care they received.	The results suggest that the key positive characteristics of care during crisis situations are intrinsically linked to the underlying values and principles of CRHTT services and can be summarized as follows: access to and availability of nurses, patients being treated as “normal” human beings, and the approach to crisis management within the context of everyday life.	Satisfaction was analyzed solely from the perspective of patients and their families. Another limitation lies in the possibility that participants may have felt pressured to provide the “correct” response. Additionally, there was difficulty in isolating views on nursing care within a service that incorporates other perspectives of care.	1. Satisfaction with nursing care; 2. Nurse professionalism; 3. Perception of nursing care; 4. Clarification of doubts; 5. Communication and therapeutic relationship; 6. Communication with the nurse; 7. Therapeutic relationship established with the nurse; 8. Nursing care environment; 9. More tolerable at home; 10. Availability.
Taylor, 2023, Chester, UK, Crisis resolution home treatment team Clinicians’ perceptions of using a recovery approach with people with a diagnosis of borderline personality disorder (BPD), *Journal of Psychiatric and Mental Health Nursing*, 30	Qualitative Study A purposive sample from a single CRHTT, where seven registered mental health nurses were interviewed.	The objectives were as follows: 1. To understand CRHTT clinicians’ perceptions on delivering recovery-oriented care; 2. To identify the barriers clinicians face that challenge the application of recovery-oriented practices; 3. To explore the opportunities that enable clinicians to apply recovery-oriented practices.	Five themes emerged: person-centered care, the timing of care, staff involvement, risk, and the stigma of borderline personality disorder (BPD) as a label.	The transient nature of interview data reflects perceptions formed now of the interview. Time constraints may have shortened the lengths of some interviews, thereby limiting the data generated. The transferability of the data is restricted due to the single-site setting and the small, homogeneous sample.	1. Person-centered care: advocated for person-centered, recovery-oriented care; 2. Timing: despite a shared view that the service focused on a person-centered approach, there was a collective understanding that acute care was a short-term intervention; 3. Inconsistent staffing: the CRHTT setup was seen as a threat to the development of therapeutic relationships that could support a recovery-oriented approach; 4. Risk: there was consensus that risk management was a central priority; 5. Stigma of borderline personality disorder (BPD): BPD was viewed differently in terms of its etiological presentation, genuineness, and reliability compared to other diagnoses, and thus was seen through a negative lens.
Begum and Riordan, 2016, Birmingham, UK, Nurses’ experiences of working in Crisis Resolution Home Treatment Teams with its additional gatekeeping responsibilities, *Journal of Psychiatric and Mental Health Nursing*, 23	Qualitative Study Six nurses working in two CRHTTs were interviewed through semi-structured interviews. The data were analyzed using thematic analysis.	The main research questions were as follows: 1. “How do nurses experience the role of gatekeeper in addition to their responsibilities as nurses working in the CRHTT?”; 2. “What factors influence their ability to effectively monitor and safeguard?”.	Using thematic analysis, four main themes emerged: 1. Gatekeeping as a specialized role; 2. Core principles of the gatekeeping role; 3. Risk management for gatekeepers on CRHTTs; 4. The future of gatekeeping. This approach provided a deep understanding of the overall experiences of nurses working on CRHTTs with the additional responsibilities of gatekeeping.	However, the study does not represent the views of all healthcare professionals, patients, carers, and family members, as it focuses more on the nursing team’s experiences. The small sample size of six nurses makes it difficult to generalize the findings.	1. Specialized nursing role: nurses in this role possess expertise, knowledge, communication skills, confidence, and autonomy, with gatekeeping being a central responsibility; 2. Mobile nursing approach: bringing hospital-level care into the home environment; 3. Less restrictive methods of care and treatment: reducing stigma, managing risks, and ensuring patient safety.

## Data Availability

All data analyzed during this study are included in this published article.
